# Validation of the German Version of the Patient Activation Measure 13 (PAM13-D) in an International Multicentre Study of Primary Care Patients

**DOI:** 10.1371/journal.pone.0074786

**Published:** 2013-09-30

**Authors:** Katja Brenk-Franz, Judith H. Hibbard, Wolfram J. Herrmann, Tobias Freund, Joachim Szecsenyi, Sima Djalali, Claudia Steurer-Stey, Andreas Sönnichsen, Fabian Tiesler, Monika Storch, Nico Schneider, Jochen Gensichen

**Affiliations:** 1 Institute of General Practice and Family Medicine, Jena University Hospital, Jena, Germany; 2 Department of Planning, Public Policy and Management and Health Policy Research Group ISE, University of Oregon, Eugene, Oregon, United States of America; 3 Institute of General Practice and Family Medicine, Otto-von-Guericke-University of Magdeburg, Magdeburg, Germany; 4 Department of General Practice and Health Services Research, University Hospital Heidelberg, Heidelberg, Germany; 5 Institute of General Practice, University of Zurich, Zuerich, Switzerland; 6 Institute of General Medicine and Family Medicine, Department of Health, Witten/Herdecke University, Witten, Germany; Queensland University of Technology, Australia

## Abstract

The patients’ active participation in their medical care is important for patients with chronic diseases. Measurements of patient activation are needed for studies and in clinical practice. This study aims to validate the Patient Activation Measure 13 (PAM13-D) in German-speaking primary care patients. This international cross-sectional multicentre study enrolled consecutively patients from primary care practices in three German-speaking countries: Germany, Austria, and Switzerland. Patients completed the PAM13-D questionnaire. General Self-Efficacy scale (GSE) was used to assess convergent validity. Furthermore Cronbach’s alpha was performed to assess internal consistency. Exploratory factor analysis was used to evaluate the underlying factor structure of the items. We included 508 patients from 16 primary care practices in the final analysis. Results were internally consistent, with a Cronbach’s alpha of 0.84. Factor analysis revealed one major underlying factor. The mean values of the PAM13-D correlated significantly (r = 0.43) with those of the GSE. The German PAM13 is a reliable and valid measure of patient activation. Thus, it may be useful in primary care clinical practice and research.

## Introduction

Chronic illnesses are of growing importance [1] especially in primary health care [2]. Active participation of patients in their own medical care is crucial to effective management of chronic conditions. Being an active and engaged patient in one’s own self-care is associated with better health outcomes [3], [4] and cost savings [5]. Activated patients also report higher quality of life and more satisfaction with care [6], [7]. Patient activation predicts health-related behaviours, including self-management, disease prevention and health-information seeking [8]. Evidence shows that specific interventions can increase patient activation [9]. Promoting active patient participation in managing their illnesses is an important task for general practitioners (GPs) [10]. Therefore, the chronic care model seeks to empower patients to take an active role in their care and supports self-management [11]. GPs need practical measures to assess patient activity and a specific measurement is needed for research and clinical practice. The Patient Activation Measure (PAM) is a reliable and valid questionnaire that measures patient activation [8]. It is also available in a 13-item short form (PAM13) [6]. It identifies four elements of patient activation (knowledge, skills, confidence, and behaviours critical for coping with a chronic illness) and suggests four levels of activation that patients reach in becoming fully engaged in managing their own health [7]. The aim of this study was to design a culturally-adapted the PAM13-D, and to validate the questionnaire in a German-speaking primary care population.

## Methods

We performed an international multicentre cross-sectional study in three German speaking countries: Germany, Austria and Switzerland. The study included adult primary care patients from general practices. Subjects completed the PAM13-D and General Self-Efficacy scale (GSE) questionnaires. We obtained ethical approval for the study from the institutional review boards of universities in each country.

### Recruitment and Data Collection

Data collection was performed by self-rating questionnaires for approximately 25 patients in each of 19 general practices in Germany, Austria, and Switzerland. We selected these practices as a convenience sample. Each practice team recruited patients for the trial. For practices to be included, their physicians needed to be specialists in general medicine or internal medicine in Germany or have an equivalent qualification in Switzerland and Austria. We collected data between September 2011 and April 2012, obtaining written informed consent from all patients prior to inclusion in the study. Patients were free to discontinue participation in the study at any time. To be eligible, a subject had to be a registered patient at the primary care practice study site and be at least 18 years old. We excluded patients with dementia, blindness, deafness, or insufficient German language skills to respond to the questions meaningfully or without informed consent. Emergency patients were also excluded. Over two practice days, health care assistants informed all eligible patients about the study and asked them if they would participate in the study. They then handed out the questionnaires with opaque envelopes to ensure blinded data entry. We provided a financial incentive of 50 Euro to each practice participating in the study.

### Translation and Cultural Adaptation

We performed the translation and cultural adaptation of the PAM13 in accordance with the methodology suggested by the World Health Organization [12]. The questionnaire was translated by a health professional, modified by an expert panel consisting of a psychologist and a physician, then back-translated by an independent native speaker and cross-checked by the same psychologist and physician. After performing a pretest on primary care patients (N = 20), we then adjusted for cultural differences in the language of the items, creating the final version by consensus.

### Measures

#### PAM13-D

The PAM13-D consists of 13 items on a Likert scale. Each item has four response categories with scores from 1 to 4: (1) strongly disagree, (2) disagree, (3) agree and (4) agree strongly. Patient activation is quantified by a sum score ranging from 13 to 52. Higher scores represent higher levels of patient activation. We included only questionnaires with answers to seven or more items in the analyses. In the case of missing data, we divided the total score by the number of completed items and multiplied by 13 to get the adjusted sum raw score. We transformed the adjusted sum raw scores into natural logarithms to better express relative distances between the scores. Then, we transformed items from the logit metric to a standardized metric ranging from 0 to 100 (0 = lowest activation level, 100 = highest activation) to compare the German results to the original data. From this analysis, it was possible to use the cut-off points suggested in the test manual to categorize patients into four levels of activation.

#### GSE

To assess convergent validity, we used the General Self-Efficacy questionnaire (GSE) [13]. The GSE is an internationally standardized one-dimensional questionnaire that measures general perceptions of self-efficacy. The construct of Perceived Self-Efficacy reflects an optimistic belief in oneself [14]. This is the belief that one can perform difficult tasks or cope with adversity in various domains of human functioning. It can be regarded as a positive resistance resource factor. The GSE consists of 10 items on a four-point Likert scale ranging from 1 to 4: (1) not at all true, (2) hardly true, (3) moderately true, and (4) exactly true. Each item measures successful coping and implies internally stable attribution of success. In samples from 23 nations, Cronbach’s alphas for the GSE ranged from.76 to.90 [13]. Most prominent health behavior theories include self-efficacy, which is a proximal and direct predictor of intention and of behavior. According to Bandura’s social cognitive theory, both an individual’s perception of his or her ability to perform an action (self-efficacy) and his or her expectations that the action will have desirable results (outcome efficacy) are important mediators of performance [15]. Therefore self-efficacy is clinically relevant as a critical ingredient of behaviour change.

### Construct Validity

Construct validity shows whether an indicator actually measures the underlying attribute. We examined the construct validity of the PAM13-D by measuring convergent validity, which assesses if the postulated dimension of the instrument correlates with other dimensions that theory suggests should be related to it. In this case, we checked convergent validity by correlating the PAM and the GSE. Using Pearson's product moment correlations, we measured the correlation of similar dimensions in these instruments that we expected to be strongly related to each other. We considered strong correlations to be over 0.60, moderate between 0.30 and 0.60, and low correlations below 0.30 [16].

### Data Analysis

To evaluate the PAM13-D questionnaire first means and standard deviations, and distribution characteristics of the items were considered. To assess the reliability Cronbach’s alpha as a characteristic size was calculated. We defined an alpha of 0.80 or higher as the acceptable value [17]–[19]. The underlying structure of the PAM13-D was evaluated by an exploratory factor analysis. Because we only expected one factor, we used a principle component analysis for factor extraction with subsequent varimax rotation [20]. The number of extracting factors was identified by performing a scree plot. If a factor has a low eigenvalue, it does not explain much of the variance in the data and may be ignored as redundant to more important factors. We defined an acceptable eigenvalue as higher than 1.5. To ensure that the scale items were relevant for principle component analysis, we performed the criteria of sampling adequacy (Kaiser-Meyer-Olkin/KMO criterion) before factor extraction, regarding a KMO criterion greater than 0.5 as a minimum for factor analysis [21] and 0.8 or higher as optimal [20]. We then calculated item-scale correlation to evaluate the relevance of single items to the overall measurement. To determine the convergent validity, we calculated the Pearson correlation coefficient between the mean sum score of the PAM13-D and the mean sum score of the GSE. We used multivariate linear regression analysis to illustrate the influences of health condition (measured with a 10 point Visual Analogue Scale) and socio-demographic variables on PAM score. Therefore, variance of the PAM score as an independent variable was modelled with the self-reported health status, adjusted for age, gender, centre and education level. An alpha level of P≤0.05 was used for tests of statistical significance. Statistical analysis was performed using IBM SPSS 20 for Windows (Chicago, IL, USA).

## Results

### Study Population

The study included a total of 508 patients (254 female). Patients’ ages ranged from 18 to 89, with a mean age of 54.7 years ±16.5 (Table 1). The self-reported health status ranged from 0 to 10, with a mean of 6.4 (SD = 2.1).

**Table 1:** Description of the study population (N = 508).

Characteristics	Subcharacteristics	n	%
Sex	Male	249	49.0
	Female	259	51.0
Age	Under 29	42	8.3
	30–39 y	49	9.6
	40–49 y	103	20.3
	50–59 y	100	19.7
	60–69 y	100	19.7
	70 and older	114	22.4
Education	Middle school	136	26.8
	Secondary modern school	192	37.8
	High school	167	32.9
	Missing	13	2.5
Study Centre	Jena (Germany)	135	26.6
	Heidelberg (Germany)	114	22.4
	Salzburg (Austria)	132	26.0
	Zürich (Switzerland)	127	25.0

### Description of the PAM13-D

Table 2 shows the means, standard deviations, and distribution characteristics (skewness and kurtosis) of the 13 items. The majority of items show ceiling effects, with a mean of 47.0%. The transformed mean of the PAM13-D score, on a scale from 0 to 100, was 68.3 (SD: 14.8).

**Table 2:** Description of the PAM13-D items (in their original English version).

Item no.	Item	Mean (SD)	Missingvalues, n (%)	Skewness (Standard Error)	Kurtosis (Standard Error)	Floor effects,N (%)	Ceiling effects, N (%)
1.	When all is said and done, I am the person who is responsiblefor managing my health condition.	3.7 (0.6)	5 (1.0)	−1.7 (0.1)	3.1 (0.2)	4 (0.8)	354 (69.7)
2.	Taking an active role in my own health care is the most importantfactor in determining my health and ability to function.	3.6 (0.6)	7 (1.4)	−1.3 (0.1)	2.1 (0.2)	5 (1.0)	306 (60.2)
3.	I am confident that I can take actions that will help preventor minimize some symptoms or problems associated withmy health condition	3.6 (0.6)	2 (0.4)	−1.3 (0.1)	1.8 (0.2)	4 (0.8)	317 (62.4)
4.	I know what each of my prescribed medications does.	3.7 (0.5)	3 (0.6)	−2.2 (0.1)	5.2 (0.2)	3 (0.6)	393 (77.4)
5.	I am confident that I can tell when I need to go get medical careand when I can handle a health problem myself.	3.4 (0.7)	2 (0.4)	−1.0 (0.1)	1.1 (0.2)	8 (1.6)	254 (50.0)
6.	I am confident I can tell my health care provider concerns I have,even when he or she does not ask.	3.6 (0.6)	2 (0.4)	−1.7 (0.1)	2.9 (0.2)	4 (0.8)	357 (70.3)
7.	I am confident that I can follow through on medical treatmentsI need to do at home.	3.0 (0.9)	9 (1.8)	−0.6 (0.1)	−0.5 (0.2)	34 (6.7)	184 (36.2)
8.	I understand the nature and causes of my health condition.	3.1 (0.8)	3 (0.6)	−0.7 (0.1)	0.3 (0.2)	22 (4.3)	166 (32.7)
9.	I know the different medical treatment options available formy health condition.	3.0 (0.9)	8 (1.6)	−0.6 (0.1)	−0.2 (0.2)	36 (7.1)	135 (26.6)
10.	I have been able to maintain the lifestyle changes for myhealth that I have made.	3.2 (0.7)	5 (1.0)	−0.5 (0.1)	−0.1 (0.2)	7 (1.4)	173 (34.1)
11.	I know how to prevent further problems with my health condition.	3.2 (0.7)	5 (1.0)	−0.6 (0.1)	0.6 (0.2)	8 (1.6)	180 (35.4)
12.	I am confident I can figure out solutions when new situationsor problems arise with my health condition.	3.0 (0.8)	3 (0.6)	−0.6 (0.1)	−0.1 (0.2)	30 (5.9)	149 (29.3)
13.	I am confident that I can maintain lifestyle changes, like dietand exercise, even during times of stress.	2.9 (0.8)	1 (0.2)	−0.3 (0.1)	−0.6 (0.2)	19 (3.7)	135 (26.6)

Abbreviations: SD, standard deviation; PAM13-D, German version of Patient Activation Measure, called the PAM13-Deutschland.

### Internal Reliability

The Cronbach’s alpha coefficient for the full PAM13-D scale of 13 items was 0.84. Using the Pearson’s correlation coefficient, the item-scale correlation ranged from 0.46 to 0.70 (p<0.01).

### Factor Analysis

**Figure 1:**
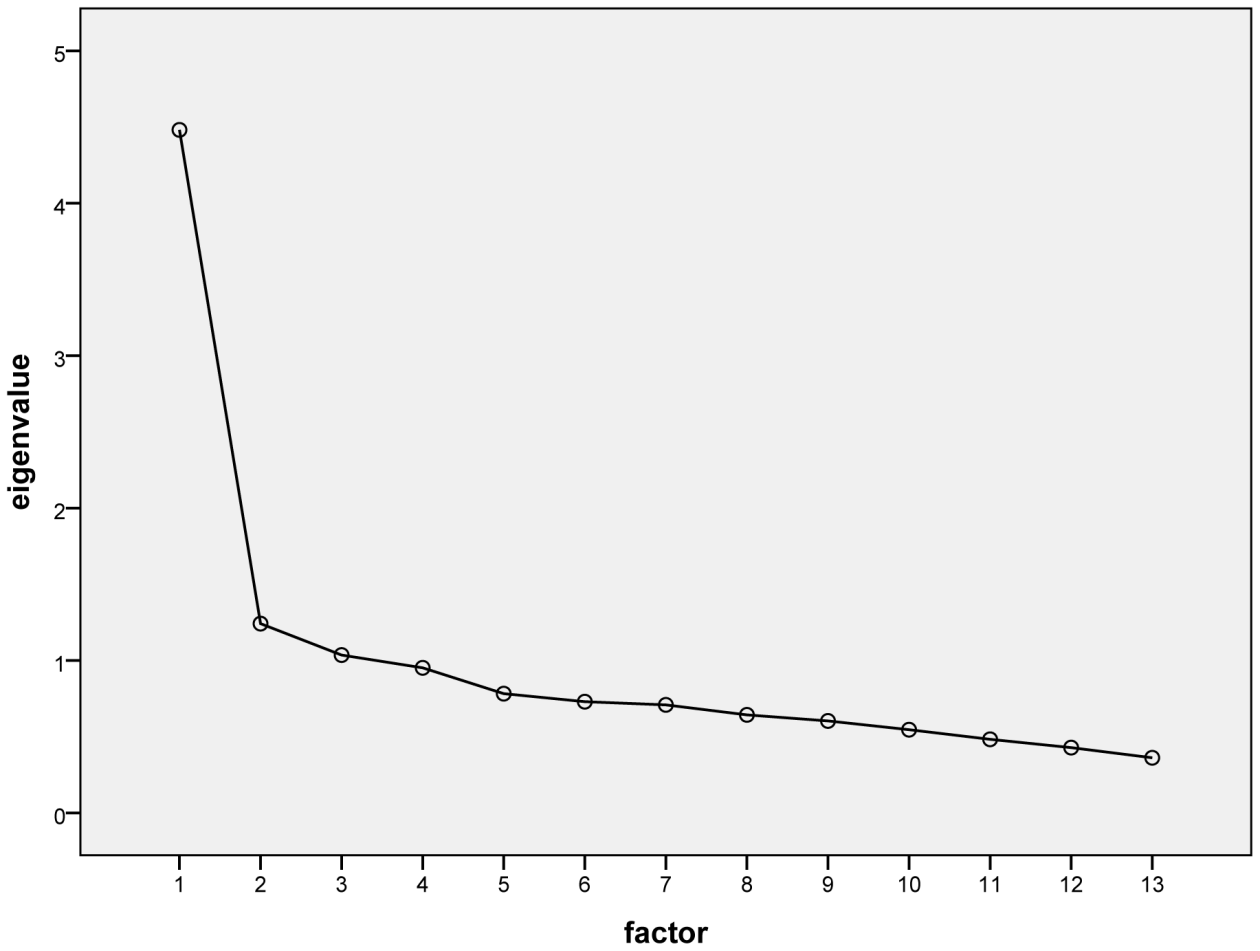
Screenplot of the PCA.

The measure of sampling adequacy showed an adequate correlation of items (KMO criterion = 0.87) which met the requirements for principal component analysis (PCA). The PCA for factor extraction with subsequent varimax rotation revealed only one dimension. The eigenvalue of the first PCA factor was 4.5, which explained 34.5% of the variance in the data. All 13 items loaded positively on this single factor (from 0.5 to 0.7). Figure 1 shows the result of this scree plot.

### Convergent Validity

The Pearson correlation coefficient between the mean sum scores of the PAM13-D and the GSE was 0.43 (p<0.01).

### Influence of Health Status

In a multivariate regression model, health status was strongly associated with PAM13-D mean sum scores (standardized b-value 1.57, P<0.001), explaining 24% of the variance (corrected R^2^ 0.087, P<0.001) if adjusted for age. Education level, gender and centre showed no significant association with PAM13-D mean sum scores.

## Discussion

We produced a standardised German translation and adaptation of the original PAM13. The forward-backward translation was successful and small conceptual modifications were made to adjust for differences in the health care systems. This was similar to other PAM validations [22]. A sample of at least 400 patients is necessary to appropriately evaluate a questionnaire [23]. Our study included 508 patients. The results of our psychometric assessment of the German version of the PAM13 replicated the findings from other translated versions [22], showing similar data quality and internal consistency.

Unlike the original PAM, the German Version showed lower internal reliability and higher patient activation scores. We suggest three factors that may account for these differences. 1) Heterogeneity of samples: Primary care samples recruited in general practice contain a wide range of patients, including both patients undergoing routine examinations and patients with multimorbidity. 2) Recruitment: Our recruitment strategy was a low-limited. Most of the patients were eligible and could be included, but patients received no financial compensation. And 3) Statistical methodology: While the authors of the original PAM suggest removing patients with extreme levels of activation from the data in order to control for responses biased by social desirability, we analyzed the complete data. Exclusion would have removed 20 patients and reduced the mean value of activation from 68.3 to 67.2 [24]. However, it is difficult to differentiate between patients reporting high levels of activation for social desirability and those who have actually achieved high levels of activation. Using a trimmed mean can lead to biased inferences [25]. Thus, we chose to analyze the data completely, following the methodological guidelines for the validation of an instrument. Our inclusion of patients with the highest possible levels of activation may explain our findings of increased activation levels, indicating ceiling effects in the German PAM13-D.

Our exploratory factor analysis of the PAM13-D indicated a homogenous factor structure with all items loading on one factor. We labeled this factor “patient activation”. The measure of sampling adequacy showed a good correlation of items, but the explained variance is not very high because there are other factors with an eigenvalue near 1. This suggests that other factors could be included, but that these are not statistically significant. The correlation between the means of the PAM13-D and the GSE was significant, providing evidence for the construct validity of the instrument. According to both our expectations and previous study results, we observed a positive association between self-reported health status and patient activation [26]–[28].

The strengths of the study include its international multicentre design in primary care settings and its systematic process of translation and cultural adaptation, as recommended by the World Health Organization [12]. Limitations of this study include cultural differences between the health systems of each study country and a possible selection bias of patients due to self-selection (resulting in ceiling effects). In addition, we had no information about the diagnoses of the patients or the presence of multimorbidity. This should be considered in future studies. Furthermore, the cross-sectional design of this study does not allow for calculation of the retest reliability because we only have one measurement point. Longitudinal validation studies should be performed before conclusions can be drawn about reproducibility of a patient’s score, test-retest reliability and measurement of intervention effects. The discriminant validity can also be determined by other instruments which could also be included in future studies. Successful evaluation of discriminant validity shows that a test of a concept is not highly correlated with other tests designed to measure theoretically different concepts [29]. Additional qualitative interviews with experts (GPs) might improve the quality of the items. Our results cannot be generalized beyond a primary care population because our sample was drawn from that specific patient population. Additional studies still need to validate the PAM13-D in other settings, in special chronic patient populations for example, in order to compare the level of activation for those groups of patients with different chronic conditions as well. Further research might analyze effects of patient activation on co-existing chronic diseases.

## Conclusions

The German Patient Activation Measure 13, the PAM13-D, appears to be a valid and reliable questionnaire to assess patient activation in primary care patients. The measure has good psychometric properties and appears to tap into the developmental character of activation. Its strong reliability supports its use as a tool for measurement in individual patients and may provide beneficial information to GPs individualizing patient care plans.
